# Three-Dimensional Surveying with Optical Sensors in Heritage Science: A Review

**DOI:** 10.3390/s26082297

**Published:** 2026-04-08

**Authors:** Emma Vannini, Alice Dal Fovo, Raffaella Fontana

**Affiliations:** 1 National Research Council—National Institute of Optics (CNR-INO), Largo E. Fermi 6, 50125 Firenze, Tuscany, Italy; emma.vannini@ino.cnr.it (E.V.); alice.dalfovo@ino.cnr.it (A.D.F.); 2 Department of Physics and Astronomy, University of Florence, Via Sansone 1, Sesto Fiorentino, 50019 Firenze, Tuscany, Italy; 3 Department of Computer Science, University of Verona, Strada le Grazie 15, 37134 Verona, Veneto, Italy

**Keywords:** 3D modelling, cultural heritage, non-contact systems, optical sensors

## Abstract

This review provides a comprehensive overview of the most adopted 3D surveying techniques in Cultural Heritage, offering practical guidance for the selection of appropriate methods when three-dimensional documentation of artworks is required. The analysis focuses on the most effective technologies for the 3D documentation of sites and objects of artistic value, with selection criteria primarily centred on non-invasiveness, given the uniqueness and cultural significance of the case studies, and the instrument flexibility, a crucial requirement for non-transportable items. A broad spectrum of 3D techniques is currently available for the multiscale diagnostic investigation of artworks, providing information at both macroscopic and microscopic levels. This review reports on the state of the art of such systems and evaluates the main characteristics of each technology in relation to its applicability in the heritage field. Particular attention is given to highlighting advantages and limitations, and to assessing performance in terms of resolution, gauge volume/area, acquisition time, and cost. In addition, the review discusses exemplary cases in which 3D methods are integrated with other analytical techniques to enable a more comprehensive understanding of the object under investigation. Finally, recent studies are examined to identify the most suitable approaches and the specific requirements for the digitization of real-world heritage assets.

## 1. Introduction

Cultural Heritage (CH) encompasses both immovable and movable assets of artistic, historical, archaeological, ethno-anthropological, archival, and bibliographic significance, as well as other items recognized as evidence of civilizational or as part of the narrative of human development. These assets hold irreplaceable historical, artistic, and cultural value, and thus demand sophisticated management strategies that span the entire lifecycle, from discovery and condition assessment to restoration, long-term monitoring, valorisation, and public accessibility.

The growing complexity of modern preservation has driven a progressive shift toward Heritage Science (HS), an interdisciplinary field founded on collaborative workflows that integrate expertise from diverse domains, including art history, archaeology, architecture, restoration, material science, engineering, chemistry, and physics. HS employs chemical and physical techniques to support restoration interventions, investigate the materials and artistic techniques used in CH assets, and assess their state of conservation. Within this framework, contemporary scientific practice increasingly prioritizes non-destructive testing (NDT), relying on non-contact approaches that enable the analysis of artworks without altering their original form.

Within this framework, 3D modelling of movable, immobile, tangible, and intangible assets has emerged as an optimal method to support the entire lifecycle of an artwork, from its discovery to public fruition. It provides the high-fidelity data necessary for digital visualization and archiving, while also informing conservation strategies. High-resolution 3D geometric data ensure that conservation decisions are grounded in measurable evidence rather than subjective interpretation. Moreover, as CH is constantly exposed to environmental threats—including climate change and natural disasters—as well as human-induced risks such as vandalism and neglect, three-dimensional documentation has become essential for creating accurate and high-fidelity digital replicas. These models support structural diagnosis and monitoring through algorithms for the automatic detection of changes across diverse contexts, from urban sites [1] to panel paintings [2], and aid restoration through virtual reconstruction. They also foster public engagement via tactile 3D prints and immersive virtual reality (VR) experiences [3,4,5], as exemplified by the MixAR mobile prototype, which overlays virtual buildings onto real ruins [6].

The surveying approach varies significantly depending on the geomorphological and physical characteristics of the artefact under investigation. At the macroscale, aerial and terrestrial methods are employed to document landscapes and extensive archaeological areas. At the mesoscale and microscale, sub-millimetric resolution techniques are required to capture statuary or minute surface details. This multiscale strategy ensures that physical features at different levels, from the overall topography of archaeological excavations to the fine craquelure of a canvas painting, are captured with appropriate spatial resolution and accuracy.

This paper reviews and compares the most recent 3D reconstruction methods with the aim of providing researchers with a comprehensive understanding of the state of the art in this field. The 3D methodologies discussed are the outcome of a systematic bibliographic review conducted to identify the most effective optical sensors and technologies for documenting cultural assets of varying scales and material characteristics. The primary objective is to offer an accessible guide for selecting the optimal scanning system, grounded in the latest scientific literature, and highlighting emerging trends and ongoing challenges. To ensure consistent evaluation criteria, certain techniques were deliberately excluded. Methods that rely on ionizing radiation to reconstruct internal structures, such as X-ray Computed Tomography (CT), or particle beam, such as Neutron Tomography, were omitted, as they do not involve optical sensors and serve fundamentally different diagnostic purposes. Similarly, although Terahertz imaging has proven effective for capturing morphological surface relief, it was excluded because the THz range does not strictly fall within the spectrum of optical radiation. Conversely, particular emphasis is placed on the integration of multiple 3D techniques and the combined use of 3D methods with 2D analytical approaches.

## 2. Methodological Approach and Literature Analysis

The literature on the application of optical sensor-based 3D surveying and modelling techniques to CH was systematically reviewed.

To define the scope of this review with precision, the PCC (Population, Concept, and Context) framework was adopted as follows:

- Population/Problem: CH assets, including artworks, museum objects, monuments, archaeological sites, and urban areas.
- Concept: application of 3D modelling methods based on optical sensors.
- Context: use of 3D techniques within the interdisciplinary field of HS for conservation, documentation, and monitoring.

**Figure 1:**
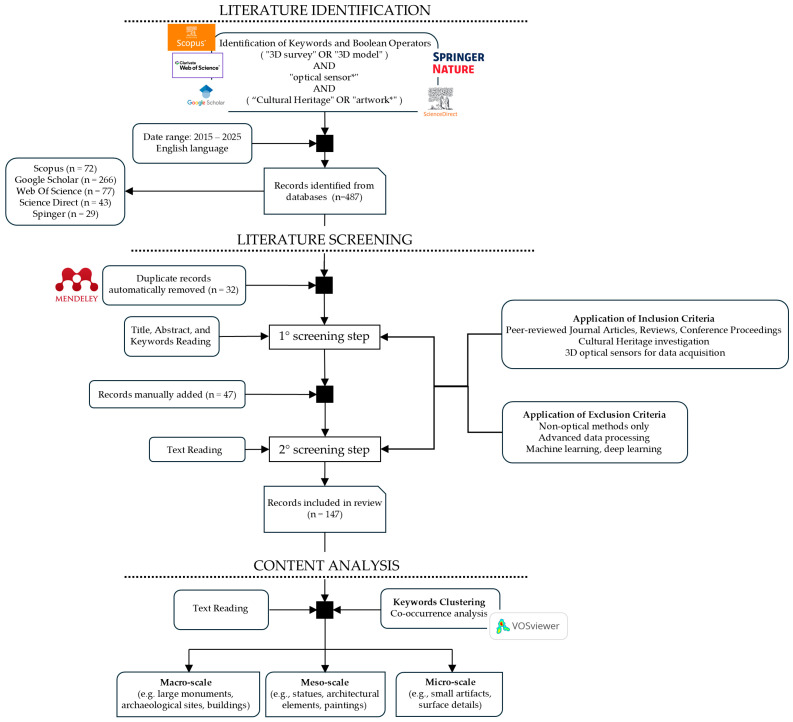
Review methodology (image by the authors).

Specific inclusion and exclusion criteria, detailed in Figure 1, were established a priori to ensure a focused and relevant selection of studies. A comprehensive literature search was conducted across five major academic databases and search engines: Scopus, Web of Science, Google Scholar, ScienceDirect, and Springer Nature. The search was restricted to works published between 2015 and 2025. All records were retrieved in November 2025. The search strategy was designed to identify studies addressing the intersection of 3D surveying, optical sensor technology, and CH. The following full search string was used:

(“3D survey” OR “3D model”) AND “optical sensor*” AND (“cultural heritage” OR “artwork*”).

Given the substantial volume of literature on 3D modelling applications in HS, a multi-stage search strategy was adopted, as summarized in Table 1. This approach allowed for a selection of studies that is both manageable and representative. The initial query, which omitted the keyword “optical sensor”, produced a large dataset, some of which was irrelevant to the scope. To focus specifically on optical techniques, “optical sensor” was applied as a mandatory filter, thereby reducing the number of results and refining the research focus. The combination of “optical sensor” with the terms “3D survey” OR “3D model” proved to be the most effective query for capturing relevant studies while maintaining the breadth necessary for a comprehensive review. The search terms successfully retrieved studies on techniques such as photogrammetry (near- and far-field), structured light scanning and laser scanning (including LiDAR and TLS), as well as 3D modelling of underwater cultural heritage, 3D thermal mapping, HBIM frameworks and virtual or augmented reality.

The identified records were then subjected to a rigorous selection process (see Figure 1). The initial search across the specified databases yielded 485 records. After 32 duplicates were removed automatically by Mendeley Reference Manager (Version 2.142.0, https://www.mendeley.com/, accessed on 25 November 2025), 453 records proceeded to the screening phase. The first stage involved a title and abstract review, which resulted in the exclusion of 281 records that were clearly outside the scope of the review. The initial database search identified only a few references related to three-dimensional optical surveying methods at the microscopic scale. Consequently, we conducted further targeted searches to improve the representation of this field of application. Given the extensive expertise of the Heritage Science Group at the National Institute of Optics (CNR-INO)—the authors’ primary affiliation—in microscale surveying, twelve studies on Optical Microprofilometry (OM) and six on Optical Coherence Tomography (OCT) were manually added to the corpus. Seven additional significant works from the CNR-INO group were included to cover Time of Flight (ToF) scanning (two studies) and Structured Light Scanning (SLS) or photogrammetry (five studies). To ensure completeness, seven further studies on macro-lens and microscope photogrammetry were incorporated.

To expand on the database results concerning the combination of 3D surveying and thermal imaging, three records focusing on the synergy between three-dimensional modelling and multi-spectral imaging were added to the six already identified. As Reflectance Transformation Imaging (RTI) was not adequately covered in the initial database search, despite being a promising 3D technique, nine relevant studies were manually included. Since the initial corpus contained three records providing a brief overview of the applications of Augmented Reality (AR) and Virtual Reality (VR) in the three-dimensional cultural heritage field, three records on Digital Twins were added to acknowledge this expanding framework.

This process resulted in the addition of 47 reports to the corpus identified through database searching, bringing the total number of reports assessed for eligibility to 221. In the second screening stage, a detailed review of the abstracts and full texts led to the exclusion of 74 additional reports that did not meet the predefined inclusion criteria. This systematic process resulted in a final corpus of 147 studies included in the review.

Table A1 in Appendix A summarizes the analysed papers, categorizing them into those retrieved from database searches and those manually added. Within each category, the works are further subdivided by technology or topic.

**Figure 2:**
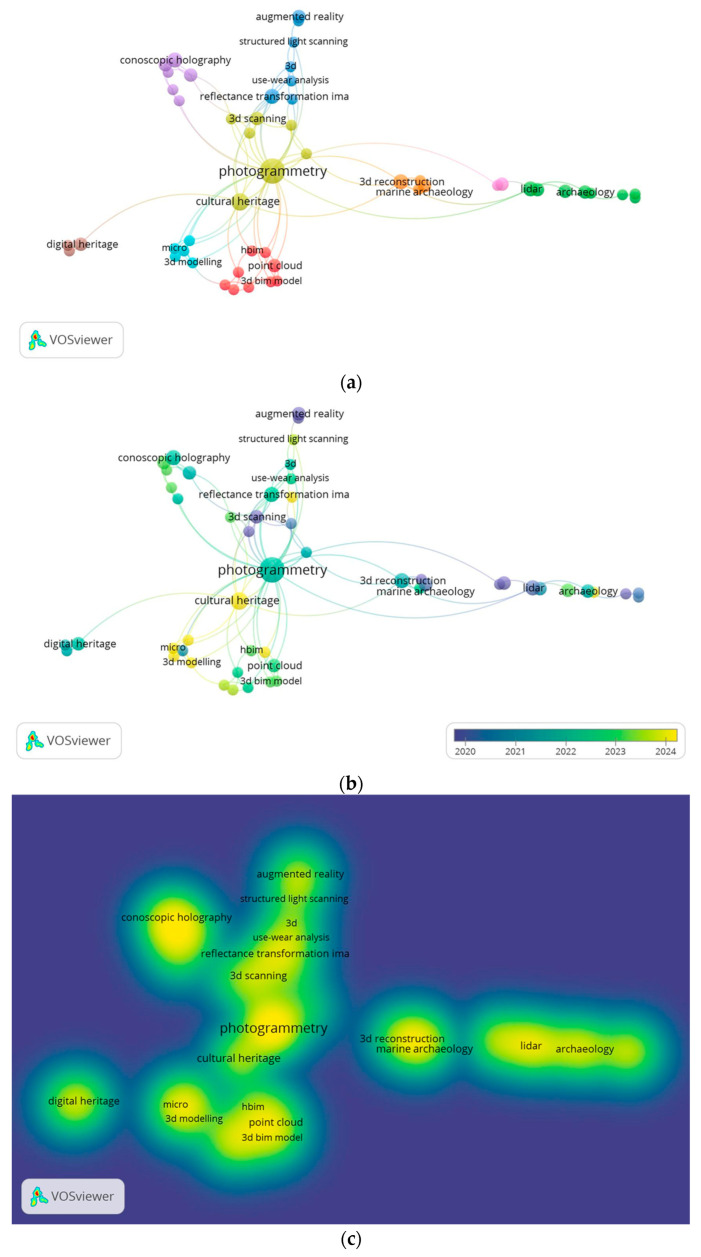
Keywords co-occurrence analysis (minimum 2 occurrences, 51 terms, 9 clusters, 111 links); (**a**) network visualization; (**b**) overlay visualization; (**c**) density visualization. Generated using VOSviewer version 1.6.20 (image by the authors).

A keyword co-occurrence analysis was performed using VOSviewer software (version 1.6.20, https://www.vosviewer.com/, accessed on 30 November 2025) to map the final corpus and identify research trends within the 147 included studies. This tool is designed to construct and visualize bibliometric networks. The analysis identified 51 key terms occurring at least twice across the body of literature. These terms were grouped into nine thematic clusters, interconnected by 111 links (Figure 2). The network visualization (Figure 2a) highlights the central concepts, with larger nodes representing more frequently occurring keywords. The overlay visualization (Figure 2b), which colour-codes words based on their average publication year, illustrates the temporal evolution of research topics. Finally, the density visualization (Figure 2c) indicates areas with a high concentration of related research.

The identification of 147 studies published over the past decade indicates that optical sensor-based 3D surveying has become firmly established in HS, supported by an active and growing research community. Photogrammetry has emerged as the predominant technique, showing the highest frequency of use. This prominence is attributable to the technique’s versatility: it is suitable for surveying large areas when digital cameras are mounted on drones or robotic platforms, and, more recently, for reconstructing fine surface details when cameras are equipped with macro lenses or replaced by digital microscopes. Laser scanning also featured prominently, owing to its high accuracy and its adaptability to both ground-based and aerial surveys. Other techniques and devices identified through the network analysis included Reflectance Transformation Imaging (RTI) and conoscopic holography. Further technologies were identified and examined through the analysis of the included reports.

The literature review and co-occurrence analysis revealed that the majority of research on 3D technology applications in CH focuses on macroscale surveys, particularly of archaeological sites and large buildings. This has led to extensive use of airborne drones equipped with various sensors, such as LIDAR scanners or digital cameras, to cover large areas. Although 3D reconstruction of medium-sized objects or detailed surface morphology is present, it remains confined to a few research groups and more specific applications. Consequently, the literature on this topic is rather limited. Approximately 60 studies focus on large-scale surveys, 35 on medium-sized object analysis, and 30 on microscale surveys (the remaining records pertain to other 3D-related topics).

The keyword “marine archaeology” emerged with a high frequency of occurrence in the density visualisation (Figure 2c), indicating that the 3D reconstruction of submerged sites, and underwater areas more broadly, has gained increasing relevance. International treaties, such as the Convention on the Protection of the Underwater Cultural Heritage, prioritize the use of non-destructive techniques and survey methods over the recovery of objects [7].

Several studies emphasize the integration of two or more 3D techniques, particularly Aerial Photogrammetry (AP) and Terrestrial Laser Scanning (TLS) (approximately 20 reports), to achieve more complete and comprehensive results. Several reviewed studies propose a multimodal approach based on the combination of 3D methods with 2D analyses (chemical, thermal, and spectral), shaping the future of HS in the development of Digital Twins for sustainable monitoring. Given the effectiveness of integrating different techniques through a multidisciplinary and synergetic approach, a dedicated section is included (Section 7).

## 3. Classification of 3D Methods

The variety of methods employed for 3D data acquisition makes a rigorous classification challenging. A primary distinction can be drawn on the basis of the sensing technology used, encompassing optical methods—such as interferometry, structured light scanning, laser scanning, and photogrammetry—as well as non-optical techniques like X-ray computed tomography [8,9]. Accordingly, measurement techniques may be grouped into three categories: laser-based methods; “shape-from-X” approaches, where X denotes variables such as structured light, silhouette, or stereo; and specialized techniques including digital holography or atomic force microscopy [10]. Alternatively, these methods are categorized into three practical workflows in [11]: terrestrial LiDAR, photogrammetry, and Simultaneous Localization and Mapping (SLAM)-based methods.

The available 3D tools can also be categorized according to their operating principle: interferometry, triangulation, and ToF. Interferometry relies on the superposition of light waves to perform high-precision measurements and is essential for microscopic surface topography in techniques such as OM and OCT. Triangulation calculates the 3D coordinates of a point based on known distances and angles between two camera positions or between a projector and a sensor. Techniques founded on this principle include photogrammetry, which uses 2D photographs taken from different angles, and RTI, a more recent development. Also based on triangulation are SLS and laser scanning, which project non-coherent light patterns or laser beams, respectively, that are distorted by the geometric shape of the surface. Finally, ToF measures the time required for a laser pulse to travel from the sensor to the object and back. This principle underpins most terrestrial and aerial laser scanners. The latter also typically employs the phase-shift principle, which computes distance by emitting a continuous, intensity-modulated light wave and comparing the phase difference between the emitted and returned beam.

Reconstruction methods can be further classified as active or passive systems. Active 3D technologies, such as laser scanning and structured light, project a coded light onto an object to measure its surface topography. In contrast, passive methods, such as digital photogrammetry, do not require a controlled light source to generate the 3D model; additional illumination is used only to enhance surface details and improve the final texture.

3D technologies are often categorised as either image-based (Image-Based Modelling, IBM) or range-based (Range-Based Modelling, RBM). Image-based methods extract coordinates from 2D images using Structure from Motion (SfM) and Dense Multi-View 3D Reconstruction (DMVR) algorithms. Range-based methods, by contrast, directly capture depth information to generate point clouds.

**Figure 3:**
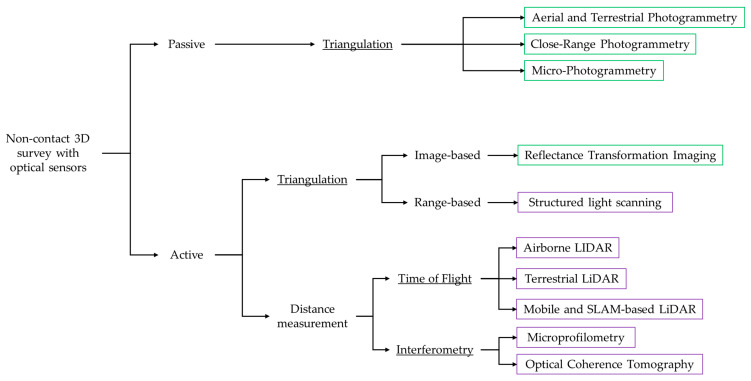
Categorisation of the 3D techniques cited in this review as active or passive according to their operating principle, which is underlined in the figure for clarity. Image-based methods are framed in green and range-based methods in violet (image by the authors).

Figure 3 summarises the techniques cited, categorised as active or passive according to their operating principles. Table 2 provides a comprehensive overview of the aforementioned 3D techniques, organized by sensing technology, operating principle, and working scale. The final column shows how the studies are distributed across the different surveying techniques and the total number of records for each technology.

Finally, a distinction can be made among capture techniques according to the size of the object, ranging from microscopic artefacts and museum objects to buildings and vast archaeological sites [10,12]. The characteristics of the artwork to be analysed are fundamental to selecting the most appropriate 3D method, given that a device’s parameters and performance are better suited to objects of a certain size [13]. In accordance with this principle and the bibliometric analysis performed (Figure 2), this review classifies 3D methodologies into three categories determined by the dimensions of the object under investigation and the distance between the object and the sensor (summarized in Table 3). The macroscale group (Section 4) includes landscapes, urban environments, architectural complexes, and underwater or archaeological sites, with stand-off distances ranging from tens of metres to several kilometres. The mesoscale group (Section 5) comprises statuary, paintings, architectural elements, and museum objects, which typically range in size from 10 cm to 2 m and are usually captured from a distance of a few metres. The microscale group (Section 6) includes small, highly detailed objects between 1 cm and 10 cm that require micrometric resolution and stand-off distances of only a few centimetres.

**Table 2:** Classification of 3D documentation techniques covered in this review. The final column indicates the section of the review dedicated to each technique.

Technique		Physical Principle	Type	Data Classification	Scale of Application	Section in the Review	Related Studies *
Photogrammetry	Arial/Terrestrial Photogrammetry (AP/TP)	Triangulation	Passive	Image-based	Macroscale	4.1	[14,15,16,17,18,19,20,21,22,23,24,25,26,27,28,29,30,31,32,33,34,35,36,37,38,39,40,41,42,43,44,45,46,47,48,49,50,51,52,53,54,55,56] (*n* = 43)
	Close-Range Photogrammetry (CRP)				Mesoscale	5.1	[16,57,58,59,60,61,62,63,64,65,66,67,68,69,70] (*n* = 15)
	Micro Photogrammetry (MP)				Microscale	6.1	[71,72,73,74,75,76,77] (*n* = 7)
Laser Scanning (LS)		Time of Flight or Triangulation	Active	Range-based	Macroscale	4.2	[11,17,20,22,23,31,33,34,35,36,37,38,39,40,41,42,43,44,45,46,47,48,51,78,79,80,81,82,83,84,85,86,87,88,89,90] (*n* = 36)
					Mesoscale	5.3	[13,58,62,67,69,91] (*n* = 6)
Structured Light Scanning (SLS)		Triangulation	Active	Range-based	Mesoscale	5.2	[13,58,62,66,68,69,92,93,94,95,96,97,98,99] (*n* = 14)
Reflectance Transformation Imaging (RTI)		Triangulation	Active	Image-based	Mesoscale	5.4	[70,100,101,102,103,104,105,106,107] (*n* = 9)
Optical Microprofilometry (OM)		Interferometry	Active	Range-based	Microscale	6.2	[13,92,108,109,110,111,112,113,114,115,116,117,118,119,120,121] (*n* = 16)
Optical Coherence Tomography (OCT)		Interferometry	Active	Range-based	Microscale	6.3	[111,122,123,124,125,126,127] (*n* = 7)

* Some studies fall into more than one section when different technologies are employed. Not listed studies cover 3D reviews, VR and AR applications comprising Digital Twins, HBIM frameworks, and 2D-3D approaches.

**Table 3:** Relationship between scaling logic, object size, and stand-off distance in 3D reconstruction.

Scale	Object Dimension	Object-to-Sensor Distance	CH Assets
Macroscale	>2 m	Tens of metres to several kilometres	Landscapes, urban environments, architectural complexes, archaeological sites
Mesoscale	10 cm to 2 m	Tens of centimetres to some metres	Statuary, paintings, archaeological findings, anthropological objects
Microscale	1 cm to 10 cm	Some centimetres	Small objects and fine details requiring micrometric resolution

## 4. Macroscale Survey

This section outlines advanced Remote Sensing (RS) methodologies employed for the large-scale documentation of CH. These techniques are classified into two main categories: Terrestrial and Aerial Photogrammetry (TP and AP) (Section 4.1), an image-based approach that derives three-dimensional data from two-dimensional photographs acquired at distances ranging up to several kilometres from the subject; and LiDAR laser scanning (Section 4.2), a range-based technique that utilizes light pulses to measure distances with high precision. The optical principles and operational modalities of each method are examined in detail, providing a clear conceptual and practical framework for their application in CH documentation. These subsections illustrate how terrestrial and aerial platforms, including robotic vehicles, Unmanned Aerial Vehicles (UAVs), and drones, can be deployed to capture data in complex urban settings and across extensive archaeological landscapes. Section 4.3 addresses the integration and fusion of these technologies for the generation of highly accurate and semantically rich 3D models, supporting applications in structural analysis and historic preservation. Finally, in light of the substantial body of scholarship on the topic, Section 4.4 is specifically devoted to the documentation of submerged CH.

### 4.1. Terrestrial and Aerial Photogrammetry (TP and AP)

Photogrammetry is a passive, image-based three-dimensional technique that derives 3D coordinates from 2D photographs captured from varying angles and positions. It relies on optical principles grounded in stereoscopy and parallax, whereby an object exhibits apparent displacement when observed from different lines of sight [14]. A photogrammetric survey is typically conducted using cost-effective and user-friendly equipment—such as Digital Single-Lens Reflex (DSLR) cameras—to acquire high-resolution images, often supplemented by an artificial lighting system to enhance texture quality.

The workflow for generating photogrammetric outputs is based on the integrated application of SfM and DMVR algorithms. The SfM algorithm estimates the spatial coordinates of homologous points using the camera’s intrinsic parameters, while DMVR identifies corresponding points across image sequences and reconstructs the acquisition geometry. In photogrammetric surveys, control points, placed either on the ground (Ground Control Points, GCPs) or directly on the surveyed object, serve as essential references for aligning and georeferencing datasets during the orientation process. The SfM pipeline begins with image acquisition, during which the target is photographed from numerous angles with substantial overlap, typically between 70% and 80%. The Bundle Block Adjustment (BBA) algorithm subsequently computes the 3D coordinates of selected key points, along with the EXIF (Exchangeable Image File Format) metadata for every photograph, thereby determining the precise position, orientation, and intrinsic camera parameters (e.g., focal length and lens distortion). This phase produces a sparse point cloud that constitutes the geometric foundation of the model. Multi-View Stereo (MVS) algorithms are then employed to generate a dense point cloud, which is scaled and referenced to a Cartesian XY coordinate system using known distances as input. The discrete data are subsequently interpolated into a continuous surface to construct a polygonal mesh. A textured, high-fidelity model is ultimately obtained by projecting photographic details (the texture) onto the mesh surface [15,57,58,59]. Photogrammetric outputs comprise both two- and three-dimensional graphic representations. Three-dimensional products include point clouds and meshes, whereas 2D outputs consist of orthophotos and Digital Surface Models (DSMs) or Digital Terrestrial Models (DTMs), the latter depicting the bare ground surface, excluding features such as vegetation or built structures.

Several software solutions support photogrammetric workflows and image processing, ranging from commercial packages such as Agisoft Metashape (version 2.0, https://www.agisoftmetashape.com/, accessed on 13 February 2026) to open-source tools including COLMAP (version 4.0.3, https://colmap.github.io/, accessed on 13 February 2026), VisualSFM (version 0.5.26, http://ccwu.me/vsfm/, accessed on 13 February 2026), MeshLab (version 2025.07, https://www.meshlab.net/, accessed on 13 February 2026), and CloudCompare (version 2.13.2, https://www.cloudcompare.org/, accessed on 13 February 2026). The 3D reconstructions generated using free and open-source software have been shown to yield results of considerable accuracy while maintaining user accessibility [16].

**Figure 4:**
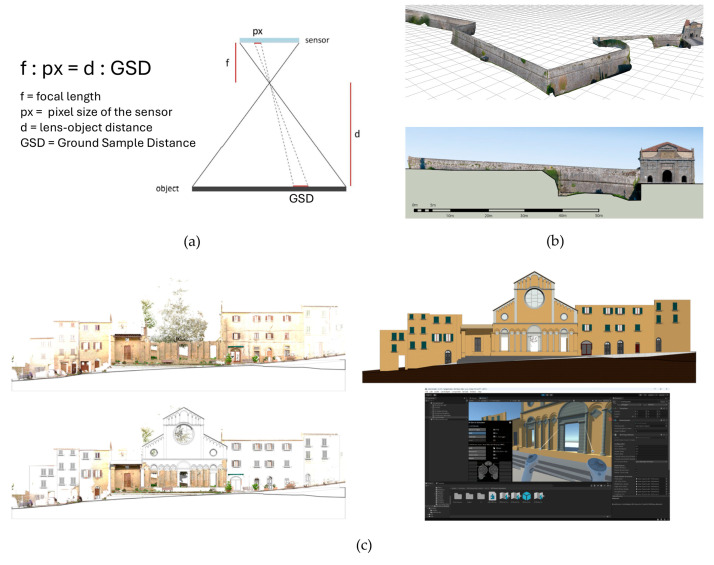
(**a**) Definition of GSD in relation to camera specifications and distance from the surveyed object. Adapted from [17]; (**b**) section and orthographic projections of the 3D model of the Venetian walls of Bergamo. Modified from [24]; (**c**) point cloud data, reconstructed HBIM model, and VR interface of the Santo Stefano Church in Volterra. Modified from [32]. The images are used under the terms and conditions of the CC BY 4.0 license (https://creativecommons.org/licenses/by/4.0/, accessed on 15 February 2026).

The achievable level of detail can be determined a priori on the basis of the device’s technical specifications—namely, sensor pixel size, focal length, and lens-to-object distance. Using the geometric relationship illustrated in Figure 4a, it is possible to calculate the object-to-device distance required to attain a given representation scale, commonly expressed as the Ground Sample Distance (GSD). The GSD is defined as the distance between the centres of two adjacent pixels onto the object surface. This parameter is of critical importance in photogrammetric surveys of large areas, where precise planning of image overlap is essential [17].

Photogrammetry can be implemented through various operational modalities, spanning terrestrial to aerial platforms.

TP predominantly employs static devices—typically tripod-mounted cameras—to acquire sequential images from close range to the subject. The recording and 3D modelling of archaeological features, including rock art panels, architectural complexes, and stone monuments, can be achieved via TP with centimetric accuracy. This approach constitutes a cost-effective alternative to high-end laser scanning systems or to labour-intensive, low-detail manual drawings, and is particularly advantageous during preliminary or rapid documentation campaigns [18]. The applicability of TP is substantiated by numerous case studies, such as the reconstruction of the city walls of Pisa [19] and the structural diagnostic survey of the vaults of Notre-Dame Cathedral, where the method was employed to assess deformations resulting from the 2019 fire. In the latter case, the authors made primary use of 2D photogrammetric products (specifically DSMs and Orthophotos) to perform a temporal comparative analysis and to detect significant deformations in critical sectors of the vaults [20].

For larger sites, AP employing UAVs enables the capture of areas that are difficult or impossible to access from the ground. UAVs, including fixed-wing and rotary-wing drones, helicopters, and multirotor platforms, can be equipped with various sensors, such as digital cameras with fixed focal length lenses, specifically configured for photogrammetric surveys [21,22]. Drone-based photogrammetry is particularly effective for surveying extensive and remote areas due to its cost efficiency and capacity for rapid data acquisition [15]. Applicative examples include the documentation of the rooftops and upper facades of the Palazzo Ducale in Gubbio [23], the extensive fortifications of the Venetian walls in Bergamo (Figure 4b) [24], the Rohan Palace and the St-Pierre-le-Jeune Catholic Church in Strasbourg [15], and ancient Greek theatres analysed for the identification of architectural features [25]. More broadly, the technique has been applied to patrimonial buildings in both preserved and dilapidated states. This mode of acquisition is frequently conducted in conjunction with TP, and, in some cases, integrated with range-based techniques such as TLS [15] (see Section 4.3).

Photogrammetry is advancing through the development of specialized application domains, including Videogrammetry. This technique exploits the growing capabilities of SLAM-based methods to derive 3D data from video sequences. SLAM algorithms reconstruct the 3D structure of an object and the position of the sensor in real time. Videogrammetry employs video frames, sampled at fixed time intervals, as input for SfM and MVS workflow, enabling the three-dimensional reconstruction of heritage scenarios [26]. Although videogrammetry offers faster data acquisition compared to laser scanning, it requires considerably more time for post-processing [27] and typically yields models of lower metric accuracy [28].

Another advanced application is retrospective photogrammetry, which utilizes archival or historical photographs to digitally reconstruct heritage sites that have been altered, damaged, or destroyed. This approach extracts three-dimensional measurements from two-dimensional, overlapping archival images to generate metrically accurate 3D models. The workflow follows the conventional SfM pipeline; however, archival film cameras lack EXIF data (i.e., digital metadata pertaining to lenses and exposure settings) and frequently exhibit significant lens distortion, which poses additional challenges for orientation and calibration [14].

Further innovations include the integration of UAV photogrammetry with depth cameras, as demonstrated in the 3D reconstruction of the Great Wall of China [29], and the application of low-cost spherical cameras capable of capturing a full 360° scene in a single image [30,31].

AP 3D datasets facilitate the development of Virtual Reality (VR) environments, particularly when integrated with Heritage Building Information Modelling (HBIM) methodologies [78]. HBIM represents an evolution of the Building Information Modelling (BIM) paradigm, specifically tailored to heritage buildings. It leverages remote survey data to construct parametric libraries of architectural structures and individual components (such as wiring, piping, windows, stairs, and doors) within a unified software environment. This approach enriches geometric models with semantic and non-geometric information, thereby supporting the management of complex historical documentation [128] and providing a dynamic platform for the documentation, diagnostic analysis, and long-term monitoring of historical assets [12]. In a recent study, the original configuration of the Santo Stefano Church in Volterra, Italy, was virtually reconstructed, enabling its architectural style to be presented through an immersive VR experience (Figure 4c). This methodology not only contributes to the safeguarding of CH but also enhances the historical value of the building and promotes sustainable tourism [32].

### 4.2. Long-Range Laser Scanning

Light Detection and Ranging (LiDAR) is an active RS technique that employs light to measure distances, enabling the rapid and accurate creation of high-precision 3D models. Suitable for operational distances ranging from a few metres to several kilometres, it is particularly effective for long-range applications.

The fundamental operating principle involves the emission of a laser beam that reflects off the surface of an object and returns to a receiver. Laser scanning systems determine the sensor–target distance according to one of three ranging principles: ToF, phase shift, or triangulation. Triangulation, however, is limited to distances of 2–3 m, so its applications are limited to medium-sized objects (see Section 5.2) [31].

The ToF principle calculates the distance based on the time elapsed between the emission of a laser pulse and the detection of its reflection from the target surface. This method enables the generation of dense, geometrically accurate point clouds.

The phase shift method employs a continuously modulated laser beam (typically infrared light at various wavelengths) and computes distance by measuring the phase difference between the emitted and reflected signals. This technique offers high accuracy over medium ranges and is frequently used for the documentation of buildings and large monuments.

Laser scanning technologies can be categorized as static or dynamic, depending on the portability of the device and the platform on which the sensor is mounted.

**Figure 5:**
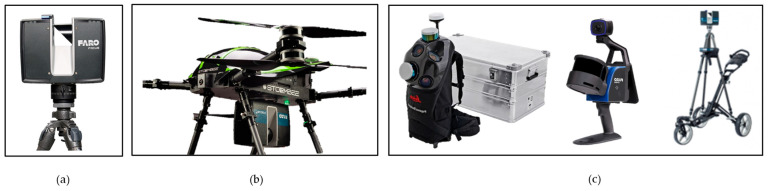
Examples of laser scanning platforms: (**a**) terrestrial (tripod-mounted) system, image by the authors; (**b**) aerial system (UAV- or drone-mounted), adapted from Geo Week News (2018) https://www.geoweeknews.com/news/a-faro-focus-on-a-uav-for-wide-area-scanning, accessed on 15 February 2026; (**c**) portable mobile mapping systems (backpack, handheld, and trolley), adapted from Line Surveying (https://line-surveying.com/surveying-equipments/leica-pegasus-backpack, accessed on 15 February 2026), Faro (https://www.faro.com/it-IT/Products/Hardware/FARO-Orbis-Mobile-Laser-Scanner, accessed on 15 February 2026), and Kwipped (https://www.kwipped.com/rentals/product/faro-focus-swift-laser-scanner-mapping-system/27291, accessed on 15 February 2026).

Ground-based (terrestrial) LiDAR (Figure 5a) involves a scanner positioned on a fixed tripod, enabling sub-centimetre accuracy in the capture of building facades, intricate architectural details, and interior spaces [79]. Further discussion is provided in Section Terrestrial Laser Scanning (TLS).

Airborne LiDAR systems (Figure 5b) are typically deployed on aircraft or UAVs for the survey of extensive heritage landscapes, archaeological sites, and large-scale structures exteriors (including vertical building facades [80], the Ming Great Wall [81], and the external topography of a cave [33]).

Mobile and SLAM-based LiDAR systems (Figure 5c) are mounted on ground vehicles, robotic platforms, or handheld by a surveyor. These systems are designed for real-time mapping of complex, confined, or inaccessible environments, and are particularly effective for documenting archaeological excavations [11], intricate interiors and subterranean built heritage, where stationary setups are often impractical [33,79,82].

The resulting point clouds are georeferenced to real-world coordinate systems through the integration of Global Navigation Satellite System (GNSS) receivers and Inertial Measurement Units (IMUs), which track the sensor’s precise position and orientation during acquisition.

More recently, Apple has integrated a LiDAR sensor into its consumer devices. This sensor enables the generation of 3D models of architectural and cultural objects of limited formal or textural complexity [83].

#### Terrestrial Laser Scanning (TLS)

TLS is a sub-category of LiDAR specifically designed for high-accuracy surveying from ground-based platforms, typically optimized for short to medium ranges (up to approximately 350 m). The measurement principle of TLS is based on either the ToF or the phase shift technology [84]. In heritage contexts, TLS is frequently preferred over TP, as it generates accurate metric 3D outputs without requiring the application of physical targets onto historical artefacts [12]. Although TLS is widely recognized for its high precision and resolution, its deployment may be constrained by equipment costs and the relative bulkiness of the instrumentation [34,35].

This methodology was employed for the structural analysis of the Notre-Dame vaults following the 2019 fire, enabling direct comparison of the 3D geometry of the vaults over time. The approach proved essential in delivering the high-precision data required for a reliable, quantitative diagnostic assessment, an outcome that could not be achieved through the initial photogrammetric survey alone [20]. Additional case studies, including Naziresha’s Mosque in Albania and the minaret of El Atik’s in Algeria, demonstrate that TLS data can reveal critical structural vulnerabilities, such as the effects of soil settlement [85] and the deformation of heritage at risk [86]. The technique is also effective in detecting structural damage in masonry constructions, including arch bridges and load-bearing walls [84].

**Figure 6:**
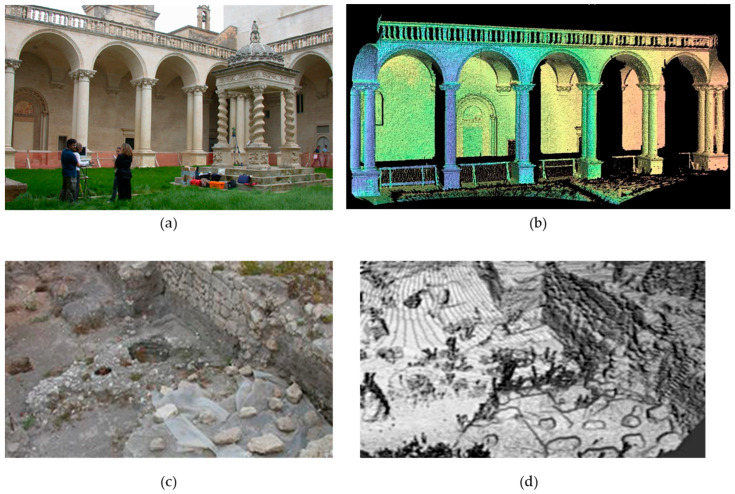
(**a**) Chiostro degli Olivetani in Lecce during the ToF laser scanner measurement campaign; (**b**) range map of the cloister; (**c**) photograph and (**d**) 3D model of the archaeological dig outside the Grotta della Poesia (image by the authors).

One of the first prototypes of a ToF TLS specifically designed for CH applications was developed in 2005 by the National Institute of Optics of CNR. It comprised a commercial distance meter mounted on a scanning system consisting of two motorised rotational stages. Its characteristics—reliability, high accuracy and resolution, low cost, and its ability to integrate with other systems—made it suitable for a wide range of applications. The scanner was employed for 3D surveys in architecture and archaeology. Examples include the Chiostro degli Olivetani (Figure 6a,b) and the archaeological excavation area outside the Grotta della Poesia (Figure 6c,d) in Lecce, Italy, the latter containing numerous archaeological artefacts such as terracotta amphorae, pots of various sizes, and oil lamps [87,88].

### 4.3. Integration of Laser Scanning and Photogrammetry in Large-Scale Contexts

The integration of multiple RS technologies facilitates the creation of detailed, accurate, and comprehensive 3D models [36,37]. Hybrid processing methodologies that combine LiDAR and photogrammetry offer significant advantages over the use of either technique in isolation [22]. In particular, the alignment and merging of TLS and UAV photogrammetric point clouds of heritage buildings enable the generation of complete three-dimensional models characterized by well-defined planar and orthogonal geometries [38]. This integrated approach also permits direct comparison between the two techniques in terms of data sensitivity, accuracy, and cost [39]. Such multi-sensor synergy—incorporating TLS, DSLR cameras, and UAVs—is especially valuable for both rural and urban heritage sites, as it supports a multi-scalar documentation strategy ranging from broad site contexts captured via aerial platforms to intricate architectural and decorative elements documented through close-range terrestrial methods [40,41].

The efficacy of these integrated techniques has been validated across diverse heritage contexts, including complex subterranean sites, historic landmarks, and architectural ensembles. Centarti et al. documented the Grotta San Michele Arcangelo by employing LiDAR-UAV for the exterior survey, SLAM-based technology for the interior spaces, and TP for high-resolution wall decorations recording [33]. Additional successful reconstructions include the Little Trianon Palace in Romania, surveyed using a UAV system equipped with a LiDAR sensor [80]; the Zamość Town Hall in Poland [42]; and the Nuraghe Sa Jua in Italy [35]. In Malaysia, the combination of drone photogrammetry and laser scanning was employed to capture the distinctive geometries and ornamental motifs of traditional Malay settlements façades within a 3D GIS environment [43]. This combined methodology has also been applied to the virtual reconstruction of ancient internal flame lighting systems: the resulting 3D model, generated through laser scanning and digital photogrammetry, enabled the simulation of lighting scenarios based on diverse historical hypotheses and the reconstruction of period luminaires [89].

Furthermore, Air–Ground Data Fusion LiDAR represents an advanced reconstruction method that employs SLAM algorithms to achieve real-time synchronization between UAVs and ground-based robotic platforms [79].

These hybrid datasets are fundamental to the Scan-to-BIM workflow. By generating integrated meshes from TLS and AP/TP, data can be imported into BIM software for rigorous structural analysis [44,45], as exemplified by the updated documentation of the Alencar theatre [46,47]. Furthermore, when combined with historical archival research, these digital tools enable the reconstruction of the structural evolution of modified buildings [48] and ultimately support the development of mixed-reality platforms [34].

### 4.4. Submerged Cultural Heritage

Oceans and seas contain a vast and largely unexplored repository of human history, designated as Underwater Cultural Heritage (UCH). This heritage encompasses deep archaeological sites [49], shipwreck remains [50,129], submerged artefacts, and inundated caves [51]. The documentation and preservation of these sites are particularly challenging due to corrosive saline environments, limited visibility, the absence of GPS signals, and the distorting effects of water on optical propagation. Both passive and active optical methods are employed for 3D reconstruction in underwater contexts. Photogrammetry is widely adopted; its principal challenge, underwater refraction, is addressed through specialized calibration techniques and refraction-aware software models [130]. In contrast, submerged laser scanning systems operate on ToF or triangulation principles and are capable of functioning at depths of up to 4000 m. Recent technological advancements, including structured light scanners, have further enhanced underwater surveying capabilities, achieving measurement accuracies as high as 0.02 mm at close range [51].

To overcome the physiological constraints of human divers, underwater sensors are typically deployed from surface vessels or integrated into robotic platforms [7,52]. Remotely Operated Vehicles (ROVs), guided in real time by surface-based operators [131], have been used for the documentation of the Cala Minnola shipwreck site [129]. Alternatively, Autonomous Underwater Vehicles (AUVs) are programmable robotic systems capable of executing missions without continuous human intervention. Although highly effective, AUVs are often limited by their considerable cost and size.

Contemporary underwater surveys increasingly exploit SLAM methods to construct three-dimensional maps of submerged environments while concurrently tracking vehicle position. Similarly, Visual Odometry (VO) estimates platform motion by tracking visual features across consecutive image frames, providing an efficient means of generating rapid 3D models [51,130].

Visualisation technologies also play a pivotal role in underwater heritage documentation: Augmented Reality (AR) interfaces assist pilots during remote vehicle manipulation, while VR enables the simulation of underwater visibility conditions for mission planning and training purposes [132].

## 5. Mesoscale Survey

This section addresses digital documentation techniques employed for the survey of mesoscale CH objects—typically ranging in size from 10 cm up to 1 or 2 m—including statues, paintings, architectural façades, archaeological and anthropological artefacts, cartographic documents, and museum collections [133]. The 3D modelling of such objects is fundamental for scientific documentation and monitoring, as well as conservation purposes. For example, the accurate geometric modelling of marble sculptures is essential for studying their dynamic response to seismic events, as demonstrated in the case of Giambologna’s statue ‘Oceanus’ [91].

Section 5.1 introduces Close-Range Photogrammetry (CRP), an image-based technique that leverages overlapping photographs to generate 3D models with high-fidelity texture reproduction. This method is notable for its accessibility and cost-effectiveness, with stand-off distances typically ranging within a few tens of metres. Section 5.2 and Section 5.3 examine Structured Light Scanning (SLS) and Laser Scanning (LS)—both active, range-based techniques that project structured light patterns (spots or fringes) onto object surfaces to capture precise geometric data with submillimetric accuracy. The discussion emphasizes the integrative potential of these methodologies, exemplified by the combination of the dimensional accuracy and precision of laser scanning with the realistic texture mapping afforded by photogrammetry.

The final subsection explores RTI, a computational photography technique developed to reveal surface details by capturing a sequence of images of an object illuminated from multiple, controlled light directions.

### 5.1. Close-Range Photogrammetry (CRP)

**Figure 7:**
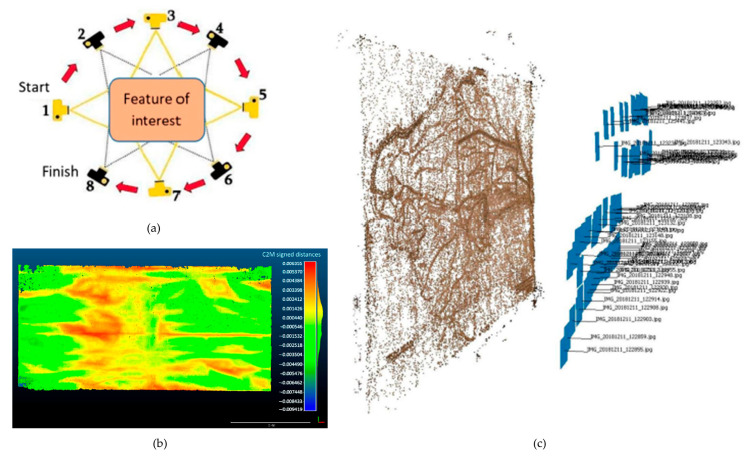
(**a**) SfM method. Adapted from [68]; (**b**) Digital Elevation Model (DEM) and (**c**) camera positions during the photogrammetric survey of a cartographic document. Modified from [53]. The images are used under the terms and conditions of the CC BY 4.0 license (https://creativecommons.org/licenses/by/4.0/, accessed on 20 February 2026).

As previously discussed (Section 4.1), photogrammetry is a RS technique that generates high-fidelity 3D models from 2D images. When the camera-to-object distance is within a few tens of metres, it is classified as CRP. This method is particularly well-suited to the survey of mesoscale artefacts requiring detailed documentation. Unlike AP, which necessitates the use of UAVs or aircraft, CRP is highly accessible and can be performed using equipment ranging from professional DSLR cameras mounted on stable tripods to modern smartphones [57,60]. The primary advantages of CRP lie in its low cost and operational flexibility, as it enables three-dimensional reconstruction without the need for expensive or highly controlled scanning environments. Image acquisition is typically performed either by moving the camera around a static object (Figure 7a) or by placing the object on a turntable that rotates in front of a fixed camera.

Controlled lighting significantly enhances the quality of CRP outputs. To capture fine surface details and mitigate shadow formation, uniform and diffuse illumination is essential. A standard professional configuration employs two external flashes or LED lamps positioned at 45° angles relative to the object [53,57,59].

CRP follows the same SfM processing pipeline outlined in Section 4.1. The workflow begins with feature extraction and feature matching across multiple overlapping images, followed by geometric verification. Known distances measured on the object—typically introduced via scale bars—are used to constrain and scale the reconstruction. Subsequent triangulation of image correspondences generates a 3D mesh, which is then subjected to a texturing phase to produce a realistic, high-fidelity model. As with AP and TP, CPR outputs encompass both 3D and 2D data products. The latter include orthophotos and Digital Elevation Models (DEMs)—raster images generated by projecting a point cloud onto a plane along an orthogonal direction.

Recent scientific literature demonstrates that CRP is highly adaptable to a variety of artefacts and research contexts, often achieving levels of precision comparable to those of more expensive technologies. Low-cost CRP employing open-source software has produced model resolutions equivalent to those obtained through active scanning systems, as evidenced in the documentation of Pre-Inca terracotta sculptures [61]. Its accessibility further renders it particularly suitable for large-scale anthropological digitization, exemplified by the 3D survey of 103 skulls conducted at the University of Florence’s Museum of Anthropology and Ethnology [62]. This methodology has also enabled the digitization of historical maps and cartographic documents with millimetre-level accuracy [53] (Figure 7b,c) and has facilitated the precise three-dimensional reconstruction and visualization of museum collections [63]. CPR proves especially valuable in contexts requiring highly accurate data, such as archaeological or architectural heritage documentation. Notable applications include the survey of ancient Roman floors [54] and the façades of historic buildings [55,56].

CRP also serves as a foundation tool for complex restoration interventions. For instance, it has provided a digital basis for the virtual reconstruction of the lost polychromy on medieval alabaster panels [64] and for the overlapping of 2D data in architectural studies [90]. Additional research has utilized orthophotos generated via photogrammetry to map pigments on wall painting fragments, thereby producing compositional maps of pictorial surfaces. In such cases, the digital model addressed the spatial constraints that prevented high-resolution photography of curved painting surfaces [65].

Recently, we employed CRP to document the restoration of a panel painting from the San Vittorino Church in Norcia (Italy), which had been severely damaged during an earthquake. The triptych required extensive structural consolidation of the wooden support. High-precision 3D models were generated both before and after the consolidation process to document the conservation state and quantify structural deformations. The photogrammetric survey was integrated with structured-light measurements to capture finer surface details [59,66,92].

Further research has explored the integration of photogrammetry with complementary techniques. By combining the high-resolution textures derived from photogrammetry with the precise geometric data obtained through LS—an approach known as Photogrammetric Texture Mapping (PTM)—researchers have developed a more robust methodology for the study of artefacts [67].

### 5.2. Structured Light Scanning (SLS)

**Figure 8:**
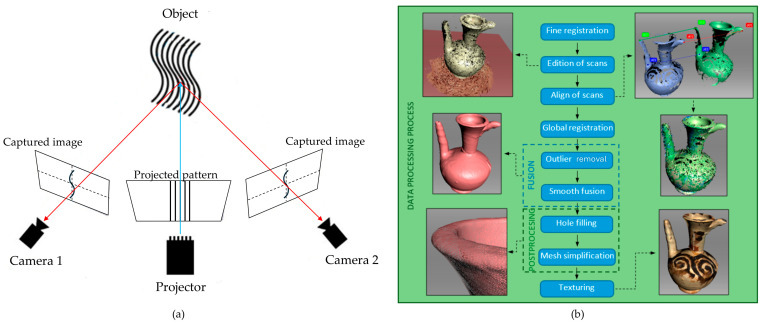
(**a**) Schematic diagram of a typical structured light system (image by the authors); (**b**) workflow of SLS data processing. Adapted from [68] under the terms and conditions of the CC BY 4.0 license (https://creativecommons.org/licenses/by/4.0/, accessed on 20 February 2026).

SLS is an active, non-contact 3D digitization technique based on the principle of triangulation. The system projects patterns of incoherent light, typically monochromatic or coloured variable-frequency fringes, onto an object’s surface. One or more cameras, positioned at a known distance from the projector, capture images of the deformation these light patterns undergo due to the object’s morphology. By analysing this distortion, the system calculates the precise 3D coordinates of numerous surface points, effectively reconstructing the object’s shape with high accuracy and sub-millimetric resolution [66,69,134] (Figure 8a).

The standard workflow for 3D digitization using SLS involves several distinct stages (Figure 8b). The process begins with the acquisition of multiple scans from various viewpoints to ensure comprehensive coverage of the object’s surface. This generates a series of individual, unaligned point clouds, which are subsequently cleaned to reduce noise. The filtered point clouds are then aligned and merged into a single coordinate system, typically employing algorithms such as the Iterative Closest Point (ICP). The registered point cloud is subsequently transformed into a continuous, structured 3D surface model, commonly referred to as a polygonal mesh. The final stage involves the application of colour (texture information) to the mesh, yielding a photorealistic digital replica of the original artefact [58,66].

**Figure 9:**
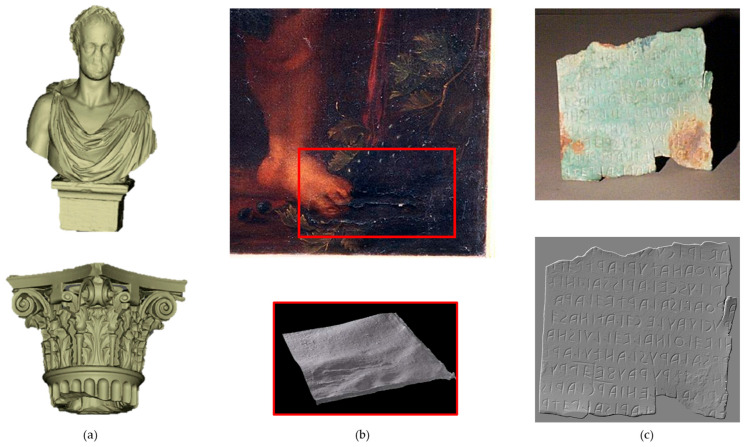
(**a**) Examples of untextured meshes of a Roman capital replica and a stone bust created with an SLS scanner (STONEX F6 SR, STONEX, Monza, Italy). Adapted from [69] under the terms and conditions of the CC BY 4.0 license (https://creativecommons.org/licenses/by/4.0/, accessed on 20 February 2026). (**b**) SLS model of the double-sided painting ‘Portrait of the dwarf Morgante’ (image by the authors). (**c**) SLS model of the bronze artefact ‘Tabula Cortonensis’ created to study the inscriptions (image by the authors).

SLS constitutes a high-precision, versatile technology, which is essential for the documentation of CH objects. By generating high-fidelity 3D models, it provides a permanent digital record that facilitates in-depth study, restoration simulations, and the creation of accurate physical replicas without jeopardizing the original artefacts (Figure 9). Its capacity for detailed geometric capture is well-documented across a diverse range of applications, including the multi-temporal analysis of panel paintings [93]; the monitoring of physical modifications in Neolithic wooden tools [94]; the analysis of digging sticks for the reconstruction of agricultural development in ancient Neolithic sites [95]; the risk-free replication of fragile and irreplaceable artefacts [96,97]; and the scanning of objects from museum exhibitions [68]. Furthermore, this technology supports anthropological research by capturing minute details of bones and fossils [62] and provides the foundational data for advanced computational workflows, such as data fusion and the automated classification of archaeological findings [58,98].

The limitations of structured light systems include challenges in accurately detecting very dark surfaces, glossy objects with reflective properties, or highly complex morphologies [10].

**Figure 10:**
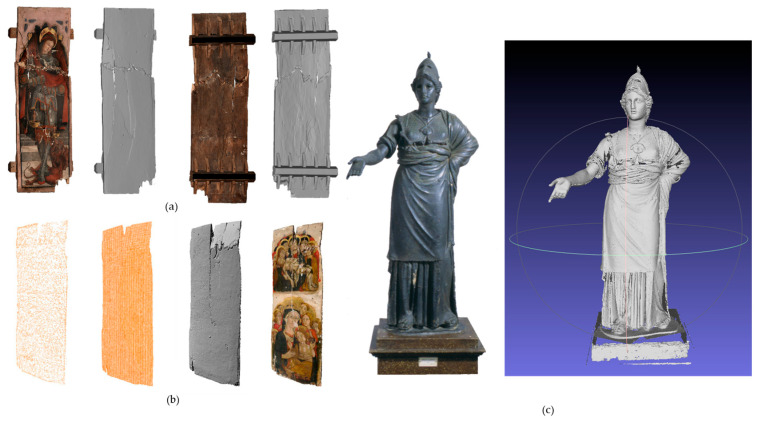
(**a**) SLS 3D models of the lateral panel of the Nocria Triptych after the earthquake; (**b**) photogrammetric 3D outputs of the central panel of the Nocria Triptych before the restoration. Modified from [66,92] under the terms and conditions of the CC BY 4.0 license (https://creativecommons.org/licenses/by/4.0/, accessed on 20 February 2026). (**c**) Photograph and 3D digital model of the Minerva statue (image by the authors).

SLS was also used for the temporal monitoring of the individual panels of the Norcia triptych (see Section 5.1 and Figure 10a,b). Similarly to the methodology adopted by Palma et al. for the ‘Adoration of the Magi’ [93], SLS was chosen for its superior spatial resolution. Figure 10a illustrates the 3D outputs of the survey, highlighting the damage following the seismic event.

While SLS relies on the projection of light patterns, other scanning systems use spot or line illumination while still operating on the principle of triangulation. An example is the high-resolution, custom-built laser scanner developed by CNR-INO. The device operates by projecting a laser light blade onto the object. When the blade intersects the surface, the light line is deformed according to the object’s geometry. The scanner moves across the object, acquiring a series of parallel profiles. A CCD camera captures the deformed profiles, thereby reconstructing the object’s physical shape. The spatial coordinates of the sampled points within these profiles are calculated through triangulation.

To scan large objects, the optical head was mounted on a motorized stage. This device was used to survey the Minerva of Arezzo, a bronze statue likely dating to the 3rd century BC and currently housed at the National Archaeological Museum in Arezzo (Figure 10c). The resulting high-resolution model enabled detailed documentation of the statue and supported a virtual restoration, allowing researchers to visualise several plausible configurations of the right arm [99].

### 5.3. Laser Scanning (LS)

Three-dimensional LS has emerged as a prominent technique for acquiring high-fidelity geometric data on mesoscale objects. The underlying principle is typically based on ToF measurement [67]; however, scanners employing phase-shift technology are also available, although these systems are primarily designed for macroscale assets such as buildings and archaeological sites (see Section 4.2).

The workflow is analogous to that of SLS and comprises point cloud acquisition, filtering, and registration (merging of multiple scans), followed by mesh generation. The main strengths of LS lie in its high geometric accuracy, resolution, and speed of acquisition. Conversely, the technology’s most significant weaknesses are its poor texture and colour fidelity, as well as its high cost. The former limitation has been addressed by combining LS with CRP, as discussed in Section 5.1 [67].

Due to its high precision in reconstructing volumetric and morphological data, this technique is highly versatile for CH applications. Reliable results can be achieved across variable geometries, surface textures, and materials, including small archaeological findings [94], wooden and anthropological artefacts [62,67], and medium-scale statuary [69].

### 5.4. Reflectance Transformation Imaging (RTI)

**Figure 11:**
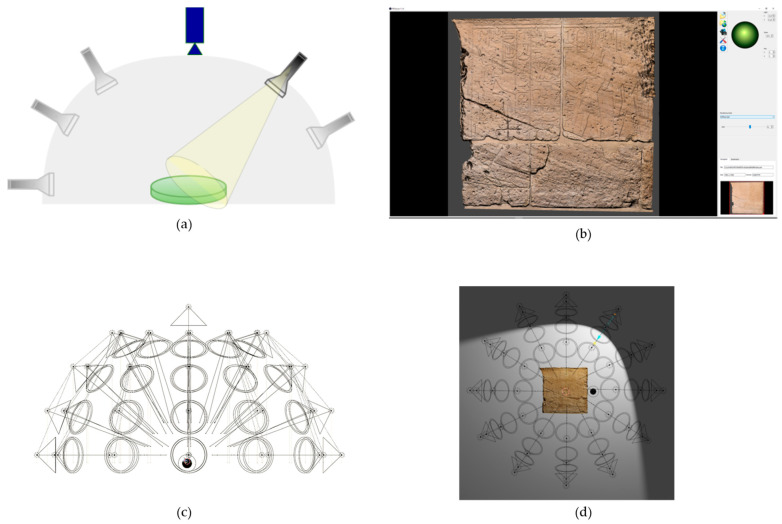
(**a**) RTI setup: the camera is fixed at the top, while the object remains stationary at the bottom. Images are captured in sequence with varying illumination directions. Adapted from [104]. (**b**) Visualization of RTI data in RTI Viewer software (Blender 5.1, http://www.blender.org/, accessed on 20 February 2026). The sphere at the top right is manipulated to simulate light originating from different directions. Adapted from [105]. V-RTI environment. (**c**) Virtual dome and (**d**) 3D model illuminated from different virtual light positions. Adapted from [105]. The images are used under the terms and conditions of the CC BY 4.0 license (https://creativecommons.org/licenses/by/4.0/, accessed on 20 February 2026).

RTI is a computational photography technique used to reveal surface details—such as faint inscriptions, worn reliefs, or tool marks—that are often imperceptible to the naked eye or with traditional photography and 3D techniques. RTI captures the reflectance properties of an object’s surface by keeping the camera stationary while recording the surface under illumination from multiple known directions (Figure 11a). This process acquires surface normals and colour information for each pixel. The primary output is an interactive digital image that enables virtual illumination of the object from any angle (Figure 11b) [70,100,101,102,103,104].

Owing to the technique’s dependence on the data acquisition process and lighting conditions, the technology has evolved from manual light positioning to automated dome systems designed for greater efficiency, repeatability, and accessibility. These devices consist of a hemispherical structure with fixed LEDs evenly distributed across its surface, allowing for the automated collection of datasets [70,102].

RTI has been successfully applied both to in situ surveys—deciphering ancient, worn inscriptions at Herculaneum that were difficult to read under conventional raking light photography [100]—and to laboratory campaigns documenting amphora stamps from Nea Paphos, Cyprus [102].

Recent developments have addressed several physical limitations of RTI, such as ambient light interference and spatial constraints. For instance, Virtual RTI (V-RTI) integrates RTI with other 3D methods: a model generated through photogrammetry is imported into a VR environment, where a synthetic dome of controllable light sources is constructed around the object, enabling interactive re-lighting and enhanced visualization of surface features. A sequence of 2D images is then captured from a fixed virtual camera, with each image illuminated by a different virtual light source (Figure 11c,d). This sequence of rendered images is subsequently processed using standard RTI software to create an interactive V-RTI file. This method was successfully applied to a bas-relief inside the chapel of Ellesiya at the Museo Egizio of Turin [105] and to ancient graffiti within the Catacombs of Naples [106].

Morita et al. [101] combined photogrammetry and RTI to produce high-quality 3D models of objects featuring microscale surface details or monochrome textures. RTI-derived high-resolution normal maps were applied as a texture to the 3D mesh generated by photogrammetry, significantly enhancing the final 3D reconstruction quality and resolution. A promising direction for future research involves applying these systems at a microscopic scale by using a stereomicroscope in place of a fixed camera, thereby enabling highly detailed analysis of artefacts [107].

## 6. Microscale Survey

This section presents specialized imaging methodologies employed for the documentation and analysis of CH artefacts at the micrometric scale, typically with dimensions ranging from 1 to 10 cm. Micro Photogrammetry (MP) (Section 6.1) utilizes high-magnification photography—such as digital cameras equipped with macro lenses or specialized digital microscopes (e.g., the Dino-Lite series, see Section Digital Microscope Photogrammetry (DMP))—to generate detailed 3D models of small objects at short stand-off distances. Optical microprofilometry (OM) (Section 6.2) employs laser interferometry to produce topographic maps of surface textures, enabling the study of fine details and surface roughness. Additionally, Optical Coherence Tomography (OCT) (Section 6.3) provides high-resolution 3D surface reconstructions and non-invasive cross-sectional views of an object’s internal layered structure.

Collectively, these technologies constitute a comprehensive suite of tools for the high-resolution documentation and analysis of archaeological findings and fine art.

### 6.1. Micro Photogrammetry (MP)

In CH and archaeology, the growing demand for high-resolution 3D documentation of small artefacts has driven the development of MP, a powerful, cost-effective, and flexible solution [71,72,73,74]. Unlike traditional photogrammetry, MP operates at stand-off distances of a few centimetres and falls within the specialised field of very close-range photogrammetry. It offers the advantages of reduced noise and enhanced metric accuracy [73]. The shorter working distance enables significantly higher magnification, facilitating the resolution of fine details for precise analysis [75].

This technique is referred to as macro-photogrammetry when it is performed using DSLR cameras equipped with macro lenses. These optical systems allow close focusing to maximise data quality and offer magnification capabilities approaching a 1:1 reproduction ratio [72].

Macro photogrammetry is founded on the principles of traditional photogrammetry and SfM algorithms. SfM software identifies and matches conjugate points in a series of overlapping 2D digital images captured at high magnification to generate a dense 3D point cloud and a textured mesh [73].

However, applying this process at a microscopic scale introduces technical challenges that are less pronounced in standard photogrammetric surveys. As optical magnification increases, the depth of field—the distance range within which objects appear acceptably sharp—diminishes significantly. Consequently, only a very thin plane of the artefact remains in focus in any single image. Furthermore, successful photogrammetric reconstruction relies on substantial image overlap for robust feature matching. Achieving this at a micro-scale is particularly difficult for objects with complex surface geometries. Additionally, uneven terrain can impede tripod placement, further complicating the acquisition process. Given that macro lens photogrammetry requires specialized photographic expertise [76], researchers are exploring more accessible methodologies. To address this challenge, the use of portable digital and USB microscopes has been proposed (see Section Digital Microscope Photogrammetry (DMP)).

#### Digital Microscope Photogrammetry (DMP)

Digital microscopes, such as the Dino-Lite series, have emerged as a popular alternative for documenting surface details, owing to their affordability and ease of use. These devices employ CCD cameras to transmit images directly to computers and typically follow a workflow involving DinoCapture 2.0 for image acquisition and Agisoft Metashape for SfM processing [73,75,77].

The portability, user-friendliness, and cost-effectiveness of these microscopes render Digital Microscope Photogrammetry (DMP) a highly competitive alternative to traditional macro lens photogrammetry. By minimizing equipment requirements, this approach streamlines the workflow and reduces the need for advanced photographic expertise.

**Figure 12:**
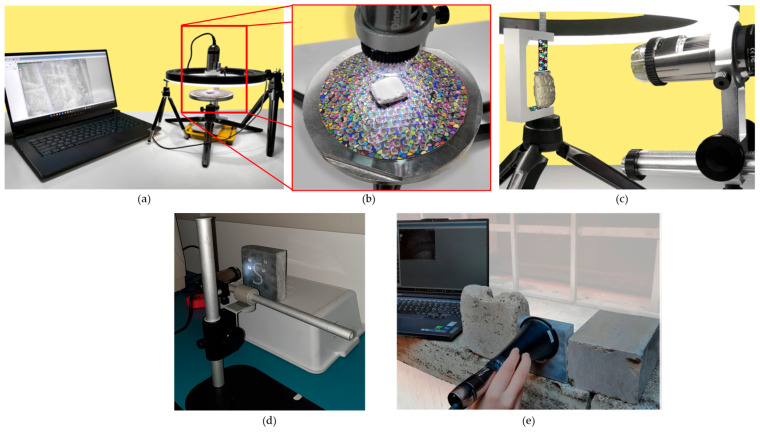
DMP setups: (**a**) support and lighting system; (**b**) 3D calibrated plate; (**c**) screw clamp setup on a rotating base. Adapted from [73]. (**d**) Microscope mounted on a stand for laboratory measurements; (**e**) protective cone for testing under natural light. Adapted from [75]. All images are used under the terms and conditions of the CC BY 4.0 license (https://creativecommons.org/licenses/by/4.0/, accessed on 22 February 2026).

However, digital microscopes share the limitations inherent to macro lenses: a narrow field of view and shallow depth of field necessitate precise focus management and extended acquisition times to ensure sufficient image overlap. Furthermore, the absence of technical and EXIF metadata in many USB microscopes continues to pose challenges in the data processing workflow [73]. Researchers have developed specialized setups to mitigate these issues, including three-dimensional calibrated plates featuring a known pattern of holes for precise scaling; advanced lighting systems designed to provide diffused, shadow-free illumination [72,73,76] (Figure 12a,b); micrometric vertical rails for focus management; rotating turntables [74,76] (Figure 12c); stable stands and protective cones to shield the lens from external light [75] (Figure 12d,e).

DMP has been employed to digitize both genuine [76] and 3D-printed [72,73] cuneiform tablets; to document mesoscopic technological traces—such as striations and use-wear marks—on archaeological findings [75,77]; to record complex metallic surfaces [74]; and to capture Roman wall painting fragments [71].

### 6.2. Optical Microprofilometry (OM)

OM is a non-invasive interferometric technique that generates three-dimensional maps of an object’s surface. The methodology achieves micrometric axial and lateral resolution and is therefore well suited to a variety of surveying applications in the CH field. These include the detection of paint detachments, the study of craquelure in pictorial layers, the calculation of surface roughness, and the analysis of micro-deformations on surfaces.

**Figure 13:**
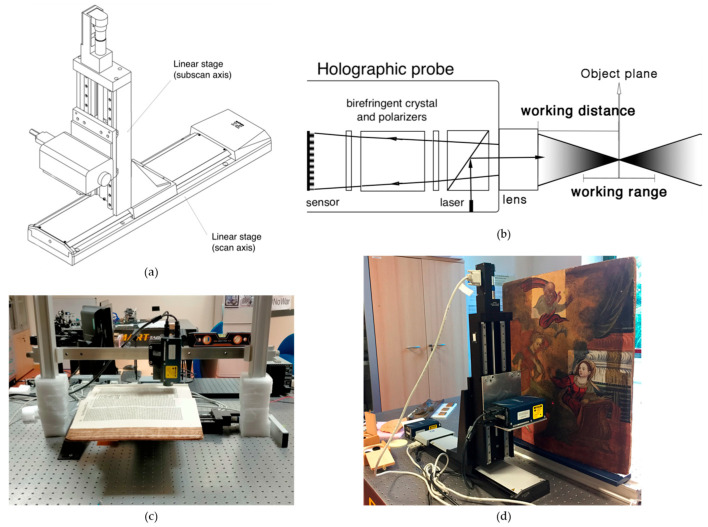
(**a**) Schematic drawings of a microprofilometer setup. (**b**) Diagram illustrating the working principle of the probe. Modified from [117]. (**c**,**d**) Application examples in real case studies. Adapted from [117,118]. The images are used under the terms and conditions of the CC BY 4.0 license (https://creativecommons.org/licenses/by/4.0/, accessed on 22 February 2026).

OM is an incoherent holographic technique based on the properties of birefringent crystals. A laser beam is directed onto the object’s surface. The backscattered light passes through an optically anisotropic crystal, which splits the beam into ordinary and extraordinary rays. Although these two beams travel along the same geometric path, they exhibit different optical path lengths. By analysing the interference pattern between the rays, the system can precisely calculate the distance from the sensor to the sampled point on the surface with micrometric accuracy [108,109] (Figure 13a,b). The primary output is a highly detailed topographic map, which can be visualized as a 3D model or a simulated raking light image to enhance surface features [66,110,111].

This technique is applicable to a wide range of materials and substrates, including corroded [112] and highly reflective metallic surfaces [113], parchment [112] (Figure 13c), drawings [114], manuscripts [115], polychrome surfaces [66,111,116] (Figure 13d), ancient tapestry [110], and archaeological findings [108,109].

Micro-surface analysis reveals fine physical traces that offer insights into an artwork’s creation and history. Microprofilometry has proven effective for the multi-temporal monitoring of surfaces under varying conditions. It has been employed to quantify the effects of environmental stress, ranging from long-term corrosion on bronze mock-ups to the rapid, dynamic response of an 11th-century parchment to microclimate fluctuations [112]. Furthermore, the technique can evaluate the impact of conservation treatments, such as surface cleaning [111], and the structural effects of wooden panel restoration on pictorial layers [92].

An additional advantage of scanning profilometry lies in the fact that the acquired data can be used to generate a detailed mesh file, enabling the production of accurate artwork replicas through 3D printing technologies [108,109,117]. Moreover, when integrated with complementary analytical methods, OM facilitates a comprehensive, multi-dimensional understanding of an artwork. The precise spatial registration of micrometre-scale height data with Multi Spectral Imaging (MSI) stacks allows researchers to correlate spectral material properties with specific physical features, such as surface roughness and layer thickness [110,114,115,118]. Additionally, the combination of microprofilometry with OCT (see Section 6.3) has proven highly effective for monitoring painting cleaning processes [111].

**Figure 14:**
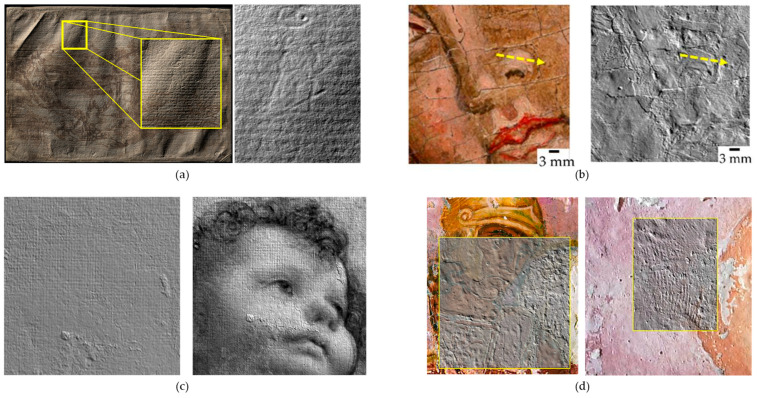
CNR-INO microprofilometry results on (**a**) Uffizi Gallery’s Drawing 8P by Leonardo da Vinci adapted from [114] under the terms and conditions of the CC BY 4.0 license (https://creativecommons.org/licenses/by/4.0/, accessed on 22 February 2026); (**b**) central panel painting of the Nocria Triptych adapted from [66] under the terms and conditions of the CC BY 4.0 license (https://creativecommons.org/licenses/by/4.0/, accessed on 22 February 2026); (**c**) simulated raking light image alone and superimposed on an IR image of a detail of Leonardo’s masterpiece ‘Madonna dei Fusi’ (image by the authors); (**d**) Amazon Sarcophagus painting (image by the authors).

The Heritage Science Group of CNR-INO has established a leading role in the application of microprofilometry to a wide range of objects, including panel, canvas, and wall paintings, paper drawings, painted parchments, tapestries, marble statues and bronze artefacts. As a representative case study, a microprofilometric survey conducted on a drawing by Leonardo da Vinci resulted in the acquisition of a high-resolution three-dimensional topographic map of the entire drawing surface, which revealed micrometric incision patterns shedding light on the transfer methodology employed by the artist (Figure 14a) [114]. Similarly, the profilometric analysis of a 14th-century triptych panel enabled the quantitative characterization of brushstrokes (Figure 14b) [66] and the detection of compass-etched haloes, *graffito* decorations, and craquelure patterns [59]. OM was successfully applied on another masterpiece by Leonardo, ‘Madonna dei Fusi’, to reveal a canvas print on the painting surface, likely resulting from a canvas used during a pictorial layer transfer operation (Figure 14c). This texture, undetectable by any other techniques but captured in the 3D model, differed from the canvas currently underlying the paint layer, as visible in the X-ray image [119]. Furthermore, significant results were obtained from the paintings of the Amazon Sarcophagus, where the analysis showed original engravings and the fingerprints left by the artists (Figure 14d).

**Figure 15:**
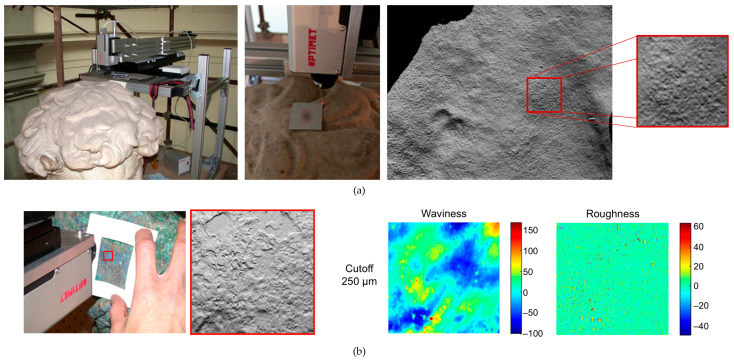
(**a**) Surface micro details of the David marble statue made by Michelangelo, analysed with microprofilometry by CNR-INO; (**b**) roughness calculation on the Minerva of Arezzo bronze statue (image by the authors).

When dealing with statues, roughness measurement is a very useful contribution to documenting surface conditions. CNR-INO has conducted extensive work in this field, notably in the analysis of Michelangelo’s David marble statue [120] and the Minerva of Arezzo bronze statue [121] (Figure 15). In the case of the David statue, roughness analysis highlighted the complex state of the marble surface, which has been significantly affected by previous restoration interventions and the long-term impact of environmental pollutants.

### 6.3. Optical Coherence Tomography (OCT)

OCT is an advanced, non-contact three-dimensional imaging technique that provides cross-sectional views and tomographic data of an object’s microstructure. It is particularly suited to the inspection of the internal structure of stratified objects that moderately absorb and scatter the probing light. As it employs low-intensity light, the technique is harmless to all types of artworks.

OCT is based on the principle of low-coherence interferometry and utilizes a broadband light source. The basic setup comprises a Michelson interferometer, which splits a beam of light into two paths: a reference arm terminating at a mirror in a known position, and a sample arm that directs light onto the object of interest. The light reflected from both arms is recombined at a detector. An interference pattern is observed when the optical path lengths of the two rays are matched within the very short coherence length of the light source. Consequently, the system can determine the depth of a reflective or scattering structure within the sample.

The coherence properties of the light source determine the axial resolution. Specifically, a broader spectral bandwidth results in a shorter coherence length and, therefore, a finer axial resolution. The bandwidth of light sources employed in OCT typically ranges from 20 to 200 nm within the 700 to 1500 nm spectral region, yielding axial resolutions of 2.0 to 20 μm in air [122]. For light sources of given bandwidths, a higher central wavelength enables greater penetration depth (up to 2–3 mm) but at the expense of reduced axial resolution.

A complete depth profile, known as an A-scan, is constructed by mapping reflectivity as a function of depth. By translating the probing beam to consecutive, adjacent positions, a two-dimensional cross-sectional image (B-scan) is generated. A three-dimensional volumetric dataset is formed from a series of B-scans, from which a transverse C-scan (en-face image) at a specific depth can be rendered. The depths recovered initially represent optical distances; correction procedures may be applied to obtain geometric distances.

The methodology for acquiring depth information has evolved significantly. The original implementation of OCT is known as Time-Domain OCT (TD-OCT). In this mode, an A-scan is acquired by physically altering the reference mirror position to match the depths of different layers within the sample. While effective, this mechanical scanning process is relatively slow. A technological advancement came with the development of Fourier-Domain OCT (FD-OCT), which enables high-speed and high-sensitivity imaging. In FD-OCT, the reference mirror remains stationary while light reflected from all depths within the sample is captured simultaneously. This may be achieved in two ways: Spectral-Domain OCT (SD-OCT), which employs a broadband light source and a spectrometer; and Swept-Source OCT (SS-OCT), which utilizes a narrow-bandwidth laser whose output frequency is rapidly swept over a wide range, coupled with a single photodetector to record the signal.

**Figure 16:**
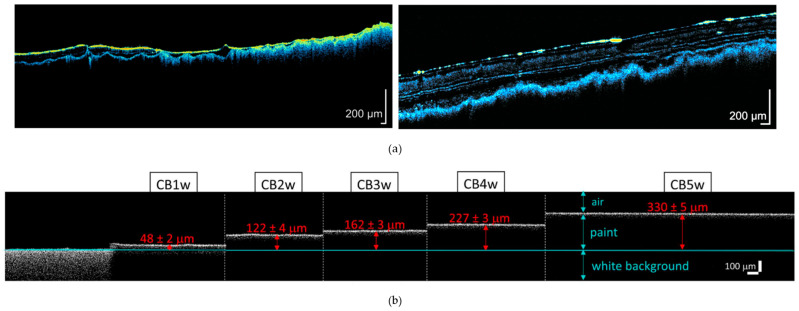
(**a**) OCT cross-sectional views of two paintings on canvas. Modified from [122]; (**b**) Composite of OCT tomograms acquired from Cobalt Blue paint applied over white backgrounds. Thickness values are calculated as the geometric distance between the air–paint and the paint–background interfaces. Adapted from [127]. The images are used under the terms and conditions of the CC BY 4.0 license (https://creativecommons.org/licenses/by/4.0/, accessed on 22 February 2026).

OCT is particularly well-suited for the analysis of paint, coatings, and other semi-transparent materials. It has been applied for the examination of varnish and paint layers [123] (Figure 16a), the monitoring of varnish cleaning processes [111], the imaging of underdrawings in paintings [124], the analysis of protective coatings on wood [125] and Ancient Egyptian faience objects [126,127], and the measure of paint layer thicknesses [127] (Figure 16b).

## 7. Multi-Modal Frameworks for Digital Replication of Heritage Assets

HS studies increasingly rely on the integration of 2D and 3D imaging data with compositional and structural information obtained from analytical techniques, enabling comprehensive documentation and characterization of CH assets. In this context, Artificial Intelligence (AI) techniques can be applied to fuse high-density 3D point clouds with imaging or spectral datasets, reducing the need for manual processing while improving the accuracy of feature detection. This multimodal approach not only supports detailed examination and preservation strategies but also provides dynamic, interactive models that serve as enduring records for monitoring, virtual restoration, and long-term conservation.

A representative example of a multimodal approach is provided by the study of two Roman silver denarii [135]. The methodology employed a thorough workflow involving hands-on inspection and optical and spectrometric techniques to document both surface details and internal structures. This non-destructive examination included photography with macro lenses, digital microscopy, RTI, and focus stacking. This approach was further complemented by 3D recording methods such as photogrammetry, LS, and SLS. To complete the analysis, physico-chemical methods, including X-ray computed microtomography (X-ray microCT), SEM/EDX, XRF mapping, and gas pycnometry, were employed.

Chemical information on the materials of an artwork can be effectively integrated into the 3D model using Multiband (MB) modelling, which combines a CRP workflow with 2D multiband imaging across different spectral bands. These include fluorescence induced by ultraviolet radiation (UVF), visible light (Vis), luminescence induced by visible light (VIL), and near-infrared (NIR) reflectography. This approach was applied to the multiband 3D reconstruction of the ‘Venus in a Bikini’ marble statue from Pompeii, which retains significant traces of its original polychromies [136]. By mapping textures acquired in the Vis, UVF, and VIL spectral ranges onto a single high-resolution mesh, the researchers created an interactive 3D model that allows users to switch between spectral bands on the same 3D geometry, enabling the exploration of different material properties and historical layers of the statue.

A similar workflow was applied to analyse the Garnier Valletti pomological collection at the University of Milan [137]. In this case, additional techniques were included: high-resolution digital radiography, pulsed thermography, XRF, FT-IR, and FORS spectroscopies. The analytical integration of imaging techniques, 3D modelling, and spectroscopic techniques provided information from the surface, subsurface, and innermost layers of the object.

**Figure 17:**
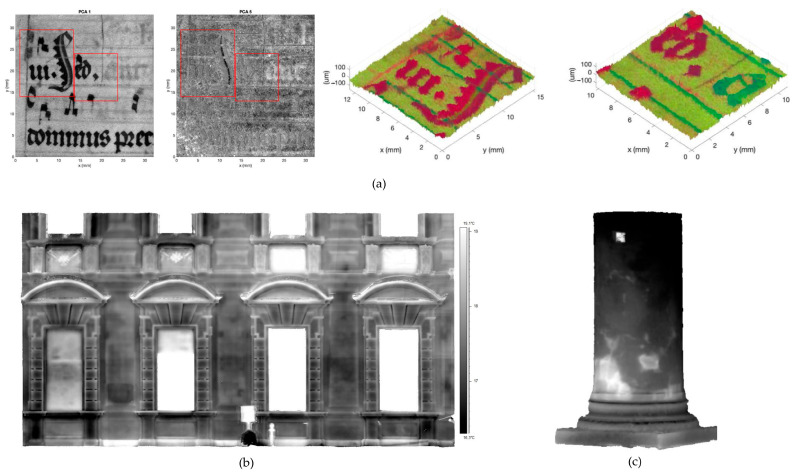
(**a**) Principal component images and surface maps of the highlighted ROI with the first, the second, and the fifth principal components mapped into RGB channels. Adapted from [115]; (**b**) View of a façade model with thermal texture and (**c**) 3D thermal model of a column base. Adapted from [138]. The images are used under the terms and conditions of the CC BY 4.0 license (https://creativecommons.org/licenses/by/4.0/, accessed on 23 February 2026).

As mentioned in Section 6.3, the spatial registration of scale height data with Multi-Spectral Imaging (MSI) stacks has also been applied at the microscopic scale. Mazzocato et al. [115] correlated images resulting from PCA of a multispectral dataset of an ancient manuscript with microsurface features (Figure 17a).

A widely adopted multi-sensor approach involves the integration of Infrared Thermography (IRT) with 3D modelling (Figure 17b,c). This combination is essential for NDT, as it enables comprehensive documentation of material distribution, plaster condition, active crack propagation, and moisture dynamics [138]. Identifying correlations between thermal anomalies and geometric deformation is particularly valuable. For example, temperature variations often reveal air gaps where structural panels have warped or detached from the underlying masonry [139].

To capture such data, thermal cameras are typically mounted on stable tripods for ground-based surveys or on UAVs for larger scale assessments. Precise alignment between the visible-spectrum model and the infrared image is critical and is commonly achieved using custom, low-cost targets that function as GCPs for multi-sensor registration.

Applications of this technique span a wide range of contexts, from the documentation of archaeological sites—including ancient theatres [140] and complex excavation landscapes [141]—to the study of architecture and historic buildings [138,139,142,143].

Ultimately, these multimodal frameworks provide the foundation for the development of Heritage Digital Twins (HDTs) [144]. Such digital replicas serve as dynamic, high-fidelity records, capturing not only the geometric and topographic features of the artefacts but also their material, spectral, and structural properties. By integrating complementary datasets from 2D and 3D imaging, spectroscopic analyses, and physico-chemical techniques, HDTs enable continuous monitoring of an object’s condition, support virtual restoration interventions, and facilitate non-invasive research. HDTs also preserve the knowledge of heritage assets in a durable and accessible format, ensuring that critical information remains available for future generations, even if the original physical objects are altered or degraded over time [145]. As demonstrated by the increasing number of case studies, including the emblematic reconstruction of Notre-Dame within the digital twin framework, the ultimate goal of HDTs is preventive conservation. This approach relies on the integration of smart sensors and AI-based technologies, enabling continuous monitoring and real-time responsiveness to environmental and anthropogenic factors that contribute to material degradation [146].

## 8. Guidelines for Selecting the Optimal Approach

Selecting a specific 3D methodology involves a calculated compromise between computational speed, accuracy, cost, and practical feasibility. This choice is guided by performance parameters and environmental constraints, which must be carefully weighed, as documented in available literature reviews [147].

Guidi et al. [12] argue that the most suitable digitization approach is primarily determined by the characteristics of the CH objects themselves, including size, material composition, state of conservation, and location. These factors impose constraints on functional parameters, such as spatial resolution, uncertainty, and the feasibility of operating in indoor or outdoor environments. Beyond logistical considerations—such as budget, timeline, and cost–benefit ratio—the primary driver for technological selection is the intended purpose of the 3D model. A fundamental distinction exists between a metric model, which provides a highly accurate virtual replica of the heritage asset or scene for precise measurement, and a non-metric 3D model, which is used for general visual representation. Although a more precise model offers greater analytical flexibility, achieving that level of accuracy often requires a significant investment in both acquisition time and processing power. Furthermore, the selection of 3D technology must align with specific data requirements of the project, whether the goal is to capture internal geometries, external topography, or surface characteristics such as colour, texture, and reflectance.

Selecting the optimal 3D digitization method requires a clear understanding of the data that each instrument or technique can capture. A wide range of 3D devices, either commercially available or developed by research groups, is suitable for art diagnostics. Instrument performance is defined by a set of technical metrics that determine its suitability for a given task. The most important among these are lateral and axial resolution (ranging from a few micrometres to centimetres), which define the level of detail captured, and accuracy, which ensures the metric reliability of the model. Meanwhile, the field of view and depth of field (from millimetres to a few kilometres) determine the gauge volume or area, while working distance and portability influence the logistic feasibility of the survey. Finally, users must balance scan efficiency (acquisition time) with the flexibility and total cost of the equipment.

Investigating an artwork usually requires both overall and detailed analyses, which in turn necessitates a multi-modal and multi-resolution approach. It is often necessary to use instruments with different gauge volumes and resolutions and subsequently integrate the results. This process is referred to as data merging, data fusion, or data integration. It involves combining multiple raw datasets to produce information that is more consistent, accurate, and useful than any individual dataset could provide on its own [66].

When applications require the integration of 3D data with complementary non-3D analytical techniques, photogrammetry is typically the preferred method. As an image-based, passive technique that does not rely on an integrated light source, photogrammetry allows for the use of various external lighting systems (such as UV, visible, or IR halogen lamps) and specialised digital sensors capable of capturing light across the ultraviolet to near-IR spectrum.

For built heritage, landscapes, and submerged archaeological sites, the priority is often scanning efficiency over extreme precision. These macroscale scenarios favour instruments with a wide FOV and working distances of several metres to cover the entire area of interest. When the budget allows, Terrestrial or Aerial Laser Scanning is preferred for its precision and targetless workflow. Alternatively, AP or TP offers a cost-efficient substitute, providing rapid data collection with centimetric accuracy. If time and financial constraints permit, a multi-resolution approach constitutes best practice.

A similar logic applies to medium-scale artefacts. When sub-millimetric resolution is paramount, active structured light scanners are the optimal choice, despite the higher equipment costs. Conversely, CRP is often employed for its flexibility and cost-effectiveness, as well as for its superior colour texture mapping capabilities. This is particularly important for generating high-resolution orthophotos of dark, glossy, or highly reflective surfaces, where laser-based systems may struggle. Artefacts often require multi-temporal monitoring to document morphological changes. If the expected deformations are minimal (e.g., millimetric deformation due to climate fluctuations), high-accuracy devices are essential.

At the microscale, the choice of technique is strictly determined by the specific data requirements. Although MP offers a flexible, cost-effective solution, it introduces significant technical challenges related to depth of field management. For reconstructing fine surface morphology, where high resolution is essential, microprofilometry represents the best approach. Although it is currently available only to specialised research groups, it can handle dark or reflective surfaces—such as gold decorations—by modulating the laser power. In contrast, modelling transparent materials poses a significant challenge for optical 3D surveying. Standard photogrammetry, SLS, and laser-based techniques, including OM, typically fail in such contexts. Consequently, these methods are often ineffective for measuring transparent or semi-transparent materials, ranging from thin varnish layers on paintings to glass objects such as unguentaria, jars, bowls, and gemmae. This limitation can be overcome through OCT. This technique generates high-resolution cross-sectional images of the investigated stratigraphy and successfully reconstructs the surfaces of glazes, protective coatings, and varnishes.

**Figure 18:**
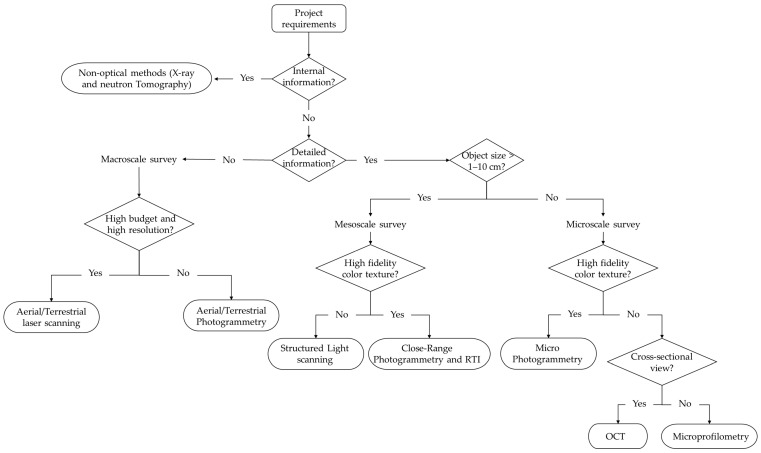
Diagram summarizing the guidelines for selecting the optimal 3D digitization approach based on object characteristics (image by the authors).

A schematic overview of the guideline for selecting the optimal approach based on object characteristics is presented in Figure 18. Table 4 summarises the performance metrics, strengths, and limitations of the discussed techniques.

## 9. Conclusions and Future Perspectives

This review aims to provide a comprehensive overview of the most used 3D techniques in the field of CH and to serve as a guide for selecting appropriate methods for creating 3D replicas of artworks. In the contemporary landscape of HS, 3D documentation has become a fundamental tool for both diagnostics and archiving. This review reports on the current state of 3D methodologies, evaluating the main characteristics of each technique with a focus on their applicability in this domain.

The literature review was rigorously conducted to ensure transparency and replicability. The PCC (Population, Concept, Context) model was employed to define the scope of the review. An initial set of 487 records identified across five major databases was subjected to a rigorous screening process using defined inclusion and exclusion criteria, yielding a final corpus of 147 studies published between 2015 and 2025. A keyword co-occurrence analysis of the literature identified the main technologies, topics, and applications across the corpus of reports.

Based on the results of bibliometric analysis, the review categorises techniques according to physical scale, taking into account the size of the object or scenario, working distances, and the intended purpose. The review covers the macroscale (landscapes and buildings), the mesoscale (statues and museum objects), and the microscale (surface morphology and internal stratigraphy). This hierarchical approach enables researchers to select tools according to specific stand-off distances—ranging from kilometres to centimetres—and the required resolution.

The primary methods for large-scale documentation are photogrammetry and laser scanning. Photogrammetry is a passive, cost-effective technique valued for its ability to capture high-fidelity textures. Conversely, TLS and LiDAR provide superior metric accuracy without the need for physical targets. Their strategic value is clearly demonstrated in structural diagnostics, where they can detect minute shifts in complex architectural geometries.

At the mesoscale, CRP offers a low-cost and highly portable solution. Laser and structured light scanners project light patterns to reconstruct object geometry, achieving submillimetric resolution. Meanwhile, RTI reveals details invisible to the naked eye by capturing surface normals under varying illumination directions.

Moving to the microscale, the review distinguishes between surface and subsurface analysis. MP employs cameras equipped with microlenses or digital microscopes to model fine surface details. OM, based on conoscopic holography, generates high-resolution topographic maps of surfaces. Finally, OCT provides cross-sectional views of internal layers, such as paint stratigraphy.

The integration of these technologies yields diverse heritage outcomes that are valuable for the diagnostic, conservation, monitoring, and fruition of artworks. The high metric accuracy of TLS enables quantitative diagnoses of structural vulnerabilities. When integrated with other methods, such as photogrammetry, it becomes possible to simultaneously survey the surrounding environment alongside intricate architectural and decorative elements. The millimetric precision of SLS and the micrometric precision of microprofilometry facilitate the monitoring of artwork deformation and changes in craquelure patterns at both the macro- and microscale.

Modern practice is further enhanced by 3D multi-band mapping, which allows geometric deformations observed through a 3D model to be correlated with the distribution of materials and thermal anomalies detected via fluorescence, infrared reflectography, and thermography.

Selecting the optimal optical sensor requires an in-depth understanding of technical trade-offs and depends on the CH object. Accordingly, a section of this review defines the guidelines for selecting the most appropriate approach. Researchers and conservators must balance portability, scan efficiency, and financial limitations against metric accuracy and resolution. Furthermore, material properties significantly influence performance; for instance, active laser-based systems often struggle with dark or glossy surfaces, making passive photogrammetry a more effective alternative in such specific contexts.

As the field matures, the state of the art is shifting towards integrated, multimodal frameworks that transform static 3D models into dynamic, “living” digital entities. AI is poised to transform this process by automating the classification of archaeological fragments and optimizing data fusion.

## Figures and Tables

**Table 1:** Summary of the multi-stage search strategy adopted.

	Number of Records Retrieved				
Search Query	Scopus	Science Direct	Web of Science	Google Scholar	Springer Nature
(“3D survey” OR “3D model”) AND (“cultural heritage” OR “artwork”)	1939	2826	797	17,000	2790
“3D” AND “optical sensor” AND (“cultural heritage” OR “works of art”)	289	85	77	674	44
(“3D survey” OR “3D model”) AND “optical sensor*” AND (“cultural heritage” OR “artwork”)	72	43	77	266	29

**Table 4:** Comparison of the performance metrics (resolution, acquisition time, cost, portability), strengths, and limitations of the discussed techniques: red = bad, blue = medium, green = good.

Technique	Resolution	Acquisition Time	Cost *	Portability	Strengths	Limitations
Photogrammetry					High texture quality	Low accuracy
LS					High accuracy	Low texture quality Highly reflective objects
SLS					High accuracy	Low texture quality Highly reflective objects
RTI					High texture quality with different light directions	Ambient light interference Spatial constraints
OM					Very high resolution	Dark and bright surfaces Lack of colour information Only nearly flat surfaces
OCT					Cross-sectional views Suitable for semi-transparent materials	Small scanned areas Lack of colour information Only nearly flat surfaces

* Considers also the difficulty in developing the instrument (as OM).

## Data Availability

No new data were created. The data created by the authors during previous research and presented in this study are available on request from the corresponding author.

## Acronyms

The following abbreviations are used in this manuscript:

AI	Artificial Intelligence
AP	Aerial Photogrammetry
AR	Augmented Reality
AUV	Autonomous Underwater Vehicle
BBA	Bundle Block Adjustment
CH	Cultural Heritage
CRP	Close-Range Photogrammetry
DEMs	Digital Elevation Models
DMP	Digital Microscope Photogrammetry
DMVR	Dense Multi-View 3D Reconstruction
DSLR	Digital Single-Lens Reflex
DSMs	Digital Surface Models
DTMs	Digital Terrestrial Models
EXIF	Exchangeable Image File Format
GCPs	Ground Control Points
GSD	Ground Sample Distance
HBIM	Heritage Building Information Modelling
HDT	Heritage Digital Twins
HS	Heritage Science
IBM	Image-Based Modelling
LIDAR	Light Detection And Ranging
LS	Laser Scanning
MP	Micro Photogrammetry
MVS	Multi-View Stereo
NDT	Non-Destructive Testing
OCT	Optical Coherence Tomography
OM	Optical Microprofilometry
RBM	Range-Based Modelling
ROV	Remotely Operated Vehicle
RS	Remote Sensing
RTI	Reflectance Transformation Imaging
SfM	Structure from Motion
SLAM	Simultaneous Localization And Mapping
SLS	Structured Light Scanning
TLS	Terrestrial Laser Scanning
ToF	Time of Flight
TP	Terrestrial Photogrammetry
UAV	Unmanned Aerial Vehicle
UCH	Underwater Cultural Heritage
VR	Virtual Reality
V-RTI	Virtual-Reflectance Transformation Imaging

## Appendix A

**Table A1:** Summary of the analysed papers, categorized into those manually added and those retrieved from database searches.

		Manually Added (*n* = 47)	Retrieved from Database Searches (*n* = 100)
Photogrammetry	Arial/Terrestrial Photogrammetry (AP/TP)	/	[14,15,16,17,18,19,20,21,22,23,24,25,26,27,28,29,30,31,32,33,34,35,36,37,38,39,40,41,42,43,44,45,46,47,48,49,50,51,52,53,54,55,56]
	Close-Range Photogrammetry (CRP)	[59,66]	[16,57,58,60,61,62,63,64,65,67,68,69,70]
	Micro Photogrammetry (MP)	[71,72,73,74,75,76,77]	/
Laser Scanning (LS)	Macroscale	[87,88]	[11,17,20,22,23,31,33,34,35,36,37,38,39,40,41,42,43,44,45,46,47,48,51,78,79,80,81,82,83,84,85,86,89,90]
	Mesoscale	[13]	[58,62,67,69,91]
Structured Light Scanning (SLS)		[13,66,92,99]	[58,62,68,69,93,94,95,96,97,98,133]
Reflectance Transformation Imaging (RTI)		[70,100,101,102,103,104,105,106,107]	/
Optical Microprofilometry (OM)		[13,92,108,109,112,113,114,115,116,117,118,119,120,121]	[110,111]
Optical Coherence Tomography (OCT)		[122,123,124,125,126,127]	[111]
3D Review		/	[1,2,7,8,9,10,11,12,130,134,147]
VR, AR, and Digital Twin application		[144,145,146]	[3,4,5,6]
HBIM		/	[19,21,32,44,45,47,128]
Multi-Modal Frameworks for Digital Replication		[135,136,137]	[138,139,140,141,142,143]
Underwater Cultural Heritage (UCH)		/	[49,50,51,52,129,130,131,132]
